# A Novel Mathematical Model of Glaucoma Pathogenesis

**DOI:** 10.5005/jp-journals-10078-1241

**Published:** 2019

**Authors:** Muneeb A Faiq, Talvir Sidhu, Rayees A Sofi, Himanshu N Singh, Rizwana Qadri, Rima Dada, Shibal Bhartiya, Meghal Gagrani, Tanuj Dada

**Affiliations:** 1,2,8,9 Dr Rajendra Prasad Centre for Ophthalmic Sciences, All India Institute of Medical Sciences, New Delhi, India; 3 J&K Health Services Department, Srinagar, Jammu and Kashmir, India; 4 Functional Genomics Unit, Institute of Genomics and Integrative Biology (CSIR), New Delhi, India; Aix-Marseille University, INSERM, TAGC, UMR 1090, Marseille, France; 5 Department of Laboratory Medicine, All India Institute of Medical Sciences, New Delhi, India; 6 Department of Anatomy, Laboratory for Molecular Reproduction and Genetics, All India Institute of Medical Sciences, New Delhi, India; 7 Department of Ophthalmology, Fortis Memorial Research Institute, Gurugram, Haryana, India

**Keywords:** Glaucoma, Glial activation, Inflammation, Mathematical modeling, Neuron–glia interaction, Retinal ganglion cell

## Abstract

**Background:**

Conventional experimental approaches to understand glaucoma etiology and pathogenesis and, consequently, predict its course of progression have not seen much success due to the involvement of numerous molecular, cellular, and other moieties. An overwhelming number of these moieties at different levels combined with numerous environmental factors further complicate the intricacy. Interaction patterns between these factors are important to understand yet difficult to probe with conservative experimental approaches.

**Methods:**

We performed a system-level analysis with mathematical modeling by developing and analyzing rate equations with respect to the cellular events in glaucoma pathogenesis. Twenty-two events were enlisted from the literature survey and were analyzed in terms of the sensitivity coefficient of retinal ganglion cells. A separate rate equation was developed for cellular stress also. The results were analyzed with respect to time, and the time course of the events with respect to various cellular moieties was analyzed.

**Results:**

Our results suggest that microglia activation is among the earliest events in glaucoma pathogenesis. This modeling method yields a wealth of useful information which may serve as an important guide to better understand glaucoma pathogenesis and design experimental approaches and also identify useful diagnostic/predictive methods and important therapeutic targets.

**Conclusion:**

We here report the first mathematical model for glaucoma pathogenesis which provides important insight into the sensitivity coefficient and glia-mediated pathology of glaucoma.

**How to cite this article:**

Faiq MA, Sidhu T, *et al.* A Novel Mathematical Model of Glaucoma Pathogenesis. J Curr Glaucoma Pract 2019; 13(1):3–8.

## INTRODUCTION

Glaucoma is an intricate disorder characterized by irreversible loss of vision ensued by retinal ganglion cell (RGC) death. It is the most common form of irreversible blindness and may possibly be the most prevalent neurodegenerative disease with approximately 70 million sufferers worldwide.^1^ Being one of the most complex neurodegenerative disorders^2–13^ (if glaucoma is classified to be one in the light of compelling evidence), the complete pathogenesis of glaucoma is still elusive. A part of the reason is that the disease is often regarded as an ocular disorder and partly many processes, events, and pathways, including but not limited to inflammatory response, intercellular signaling, oxidative stress, apoptosis, synaptic transmission, protein degradation and misfolding, taupathy, amyloidopathy, and genetics, have a role to play in its etiopathogenesis. In addition to this, there are also certain anatomical reasons which make RGCs susceptible to selective demise in glaucoma. Reasons include the unique anatomical structure of the optic nerve head,^14^ translaminar pressure difference, trabecular meshwork cellular architecture, etc. Owing to its molecular and cellular intricacies, mainstream investigations have not been successful in unraveling its etiopathomechanism. As a consequence, glaucoma still remains incurable and poorly managed progressive optic neuropathy.

With regard to the complex interplay of events and processes in glaucoma etiology and progression, we believe that a system-level approach may give some important insights into the nature of the disease and may guide through the development of possible therapeutic interventions. This is because of the fact that mathematical modeling approaches have already been undertaken in many neurodegenerative diseases including Alzheimer's disease (AD)^15^ and Parkinson's disease (PD).^16^ Mathematical modeling yields important insights^17^ into disorders involving multiple pathways which makes glaucoma a suitable candidate for the said approach.

Neuroglia interaction mediated through inflammation is one of the common denominators of pathways involved in glaucoma pathogenesis.^18,19^ Based on the interesting studies on AD,^4^ we thought to establish a mathematical model of the neuroglia interaction in glaucoma and combine it with RGC stress (an umbrella term to describe various pathological processes other than those of glial origin). To the best of our knowledge, no organized mathematical modeling approach has been reported in glaucoma pathogenesis that would aim at investigating the whole arena of cross talk between RGCs and glia. Here we report a novel mathematical approach to examine the network of cellular events and neuron–glia interactions. This approach yields vital insights into its pathogenesis and immediately suggests remedial measures to ameliorate this disorder.

## METHODS

We did comprehensive data mining (of cellular events) by employing a literature search of databases, search engines, meta-analyses, repositories, individual studies, reviews, and other online sources. The search terms used were various subsets/permutations of the following terms: apoptosis, astroglia, glaucoma, glia, inflammation, microglia, Müller cells, neuropathy, optic nerve head, optic neuropathy, photoreceptors, retina, retinal ganglion cell, and retinal neurodegeneration. The processes so mined were classified (on the basis of molecular pathways and cellular pathways) to develop a list of cellular events/processes that take place in the natural history of glaucoma etiopathology. Both proapoptotic (pathogenic) as well as antiapoptotic (homeostatic) events were enlisted. The tools to generate the list of cellular events were disgenet, genecards, metacore, genespring, cytoscape, netpath, cell collective, and E-cell. A simulation network of the important events was constructed from this list. This generated a list with a total number of 22 cellular events which were annotated as homeostatic/physiological and pathogenic (depending on their anti- or proapoptotic nature, respectively).

**Fig. 1:**
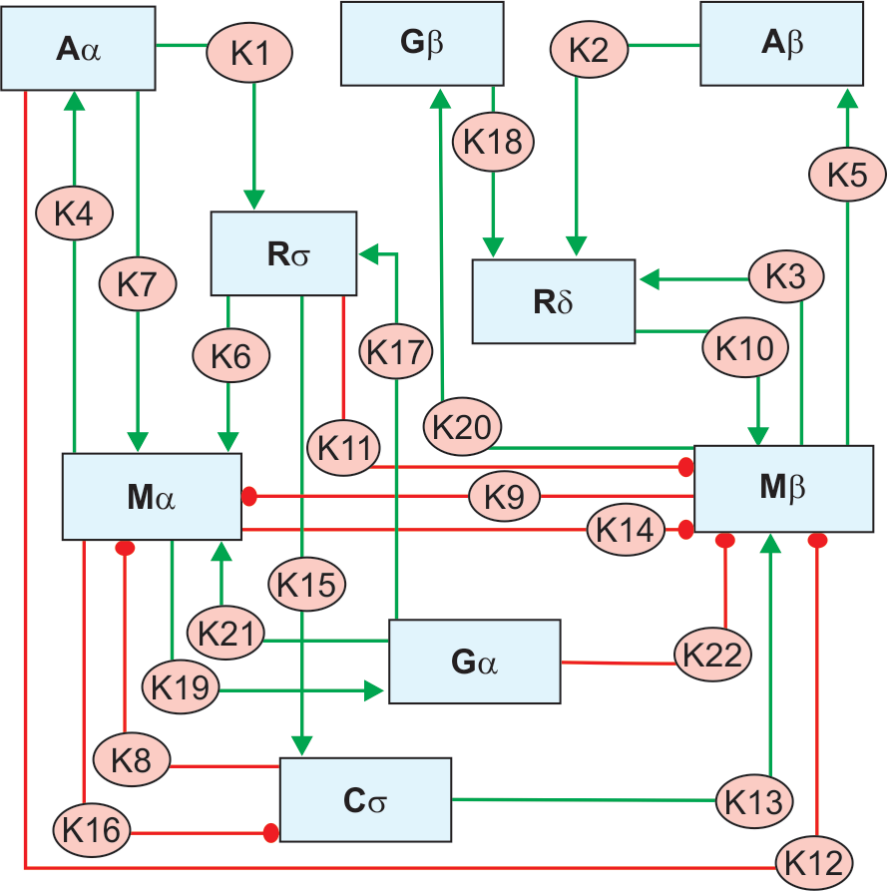
A complete overview of the cellular processes in glaucoma pathogenesis putting forth our 22-pathway hypothesis for glaucoma

Our proposition, therefore, leads to a 22 cellular pathway hypothesis for glaucoma involving eight species (state specific) of cells. We described each cell species in a particular state. Resting and activated cells of the same type were enlisted differently as they serve different functions in different states. The transformation of one cell type to another was treated as a change with a certain rate and dynamics represented by the rate constant of that change (denoted as *k_i_*). Two end points were set for each change in our model. This way all the 22 pathways/events were represented by a change with a definable rate constant. Such changes were subjected to differential analysis. The species of cells/cellular states are as follows: surviving RGCs (Rσ), dying RGCs (Rδ), quiescent astroglia (Aα), proliferative astroglia (Aβ), proinflammatory microglia (Mβ), antiinflammatory microglia (Mα), quiescent Müller cells (Gα), reactive Muller cells (Gβ), and one additional stress denominator (Cσ) representing inflammation, oxidative stress, and other proapoptotic stressors (Fig. 1).

Each of the 22 events was described by its own kinetic parameter (denoted as *k*_1_ to *k*_22_). Some of these processes are proglaucomatous and some are antiglaucomatous (as indicated in the list). These 22 pathways are enlisted as follows. Also, the nature of each process is mentioned in the parenthesis:

- Aα → Rσ; *k*_1_: (Homeostatic)
- Aβ → Rδ; *k*_2_: (Homeostatic)
- Mβ → Rδ; *k*_3_: (Homeostatic)
- Mα → Aα; *k*_4_: (Homeostatic)
- Mβ → Aβ; *k*_5_: (Homeostatic)
- Rσ → Mα; *k*_6_: (Homeostatic)
- Aα → Mα; *k*_7_: (Homeostatic)
- Cσ → Mα; *k*_8_: (Pathogenic)
- Mβ → Mα; *k*_9_: (Pathogenic)
- Rδ → Mβ; *k*_10_: (Homeostatic)
- Rσ → Mβ; *k*_11_: (Pathogenic)
- Aα → Mβ; *k*_12_: (Pathogenic)
- Cσ → Mβ; *k*_13_: (Homeostatic)
- Mα → Mβ; *k*_14_: (Pathogenic)
- Rσ → Cσ; *k*_15_: (Homeostatic)
- Mα → Cσ; *k*_16_: (Pathogenic)
- Gα → Rσ; *k*_17_: (Homeostatic)
- Gβ → Rδ; *k*_18_: (Homeostatic)
- Mα → Gα; *k*_19_: (Homeostatic)
- Mβ → Gβ; *k*_20_: (Homeostatic)
- Gα → Mα; *k*_21_: (Homeostatic)
- Gα → Mβ; *k*_22_: (Pathogenic)

After enlisting the cellular processes in glaucomatous tissue, two outcome objectives were set, *viz.*, RGC survival (Rσ) and RGC death (Rδ) as the final end points of the events. The relative population of Rσ and Rδ was correlated with glaucoma staging. The rate of increase of the Rδ/Rσ ratio was therefore indicative of the rate of glaucoma progression. For this, a sensitivity coefficient for the RGC population was identified as:

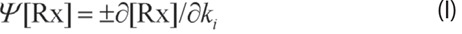


x = surviving or dead RGC. Here *Ψ*[Rx] is the sensitivity of RGCs in response to cell stress (Cσ) and *k_i_* is the kinetic parameter of any event in focus. The comprehensive network of events relevant to glaucoma identified as 22 significant cellular processes was then used in conjunction with Clarke's one-hit model of neuronal cell death^20^ which leads to the derivation of 7 rate equations for the various cell populations. The equations are:


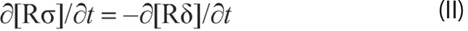



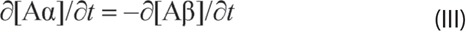



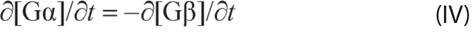



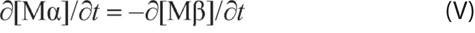



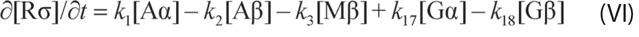



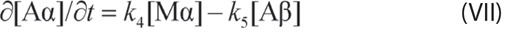



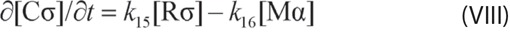


Analyses of the rate equations were done as per the protocol described by Puri and Li.^15^

Additionally, a mathematical equation was derived for cellular stress (representative of inflammation, taupathy, and various factors other than glial interactions) also. The equation was described by the rate of diffusion of inflammatory factors in the cellular milieu.


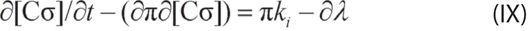


where “π” is the production of inflammatory moieties, “λ” is the degradation of the inflammatory moieties, and “∂π” is the diffusion coefficient.

Sensitivity analysis was carried out by tracing the production and removal rates of the stressors mentioned in 22 pathway events. Sensitivity analysis was followed by Latin hypercube sampling to generate the sample of parameter values from the multidimensional distribution of glaucoma pathogenesis to be followed in the *in silico* experimentation. Partial rank correlation coefficients (PRCC) and *p* values were estimated for neuronal density (density of live RGCs) and inflammation buildup for a time period of 20 years. A *p* value of less than 0.01 was considered to be significant. A positive PRCC was understood as a positive correlation indicating an increase in the RGCs and *vice versa*.

The differential equations were derived by utilizing the unknown function of one complex variable (in our case, the state of a particular cell type, e.g., from quiescent microglia to activated microglia or from surviving RGC to dead RGC), its derivatives, and some given functions of the variable. The cell state influenced by any other cellular moiety was identified as a dependent variable whereas a cellular state that is not affected by the other cell species was denoted as an independent variable. The network contained multivariable functions and their partial derivatives. The 22 different cellular events were formalized in the differential equations described above which represent a multidimensional system that can be described as stochastic partial differential equations.

**Table 1:** The cell population and the amount of cell stress (in terms of stressor moieties or units) at time *T* = 0 and the value of *Ψ*[Rx] = ±d[Rx]/d*k_i_* at *T* = *X* years which ranges from 1 to 20

*Value*	*Rσ(0)*	*Rδ(0)*	*Aα(0)*	*Aβ(0)*	*Mβ(0)*	*Mα(0)*	*Cσ(0)*	*Gα(0)*	*Gβ(0)*
	*10^4^*	*10^2^*	*10^5^*	*10^3^*	*10^3^*	*10^5^*	*10^3^*	*10^5^*	*10^3^*
*Ψ*[Rσ]	1	0	0	0	−0.2	0	0	0	0
*Ψ*[Rδ]	0	1	0	0	0.2	0	0	0	0
*Ψ*[Aα]	0	0	1	0	−0.2	0	0	0	0
*Ψ*[Aβ]	0	0	0	1	0.2	0	0	0	0
*Ψ*[Mβ]	0	0.3	0	0	1	0	0	0	0
*Ψ*[Mα]	0	−0.3	0	0	−0.2	1	0	0	0
*Ψ*[Cσ]	1	0	0	0	−0.2	0	1	0	0
*Ψ*[Gα]	0	0	1	0	−0.2	0	0	1	0
*Ψ*[Gβ]	0	0	0	1	0.2	0	0	0	1

The values of the rate constants (*k_i_*) associated with the 22 different events were arrived at by estimating the sensitivities (*Ψ_i_*) of the surviving (Rσ) and dead RGC (Rδ) populations to variations in the values of *k_i_*. Determination of the sensitivity coefficient *Ψ*(*R*_i, i_=σ_,_δ) = ∂*R*_i_/∂*k_i_* estimated for a 20-year duration was done for ±3 percent perturbations in each *k_i_* value. It is obvious; higher the susceptibility of a particular cell population to change, higher the value of *Ψ*(*R*_i, i_=σ_,_δ). The sign of *Ψ*(*R*_i, i_=σ_,_δ) determines the increase or decrease in the cell population. Literature indicates that the dominant pathway is Aα → Mβ; hence, it was assumed that it is the fastest. So we arbitrarily set its relative value at 1 per year so that other values are determined in relation to this value.

## RESULTS

**Fig. 2:**
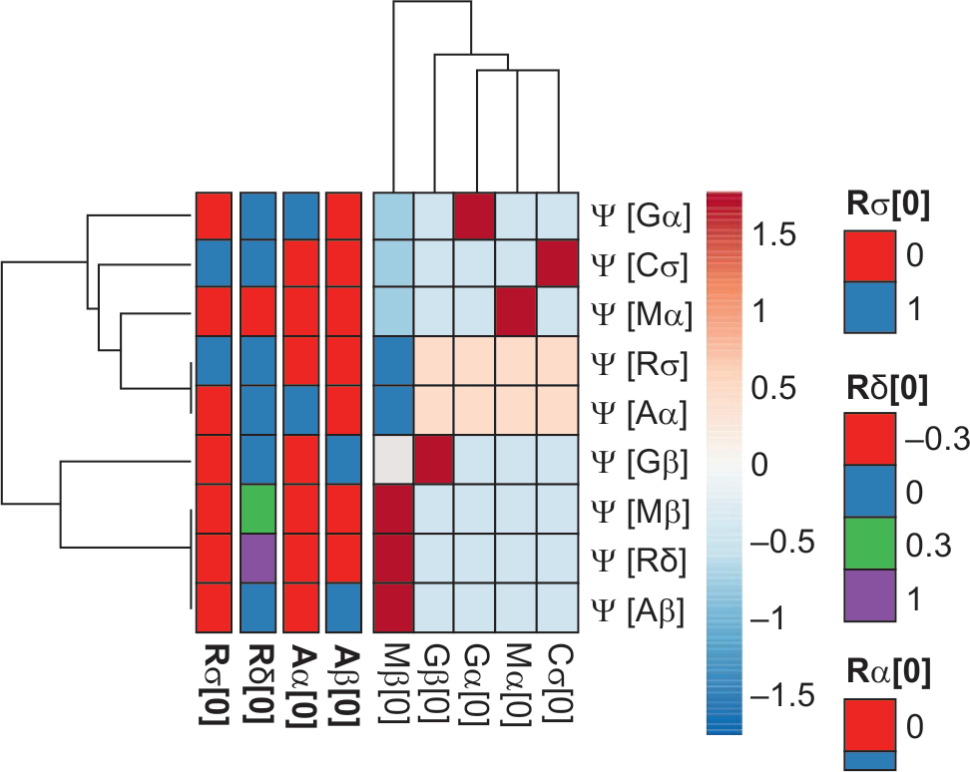
Heatmap of the sensitivities and effects of each cell species and stressors on each other of the mathematical data. This heatmap shows that the microglia have an effective interaction with RGCs. The clustering also demonstrates significant correlation coefficients of various cell species

These equations describe the progress of glaucomatous optic neuropathy in terms of the relative number of surviving and dead (including the ones cleared by various debris clearance processes) RGCs. Establishing the sensitivity prior to kinetics building in this model gives a robust estimation of the physiology to pathology to cellular demise kinetics of all the major cell populations relevant to glaucoma. A holistic overview of all these sensitivities is shown in Table 1 in which the initial estimate of cell populations and the magnitude of cellular stressors in a given retinal volume (in certain orders of magnitude) are traced in a temporal fashion for *t* = 20 years. Figure 2 demonstrates the sensitivities and effects of each cell species and stressors on each other. The figure also shows that the microglia have an effective interaction with RGCs. The clustering also demonstrates significant correlation coefficients of various cell species.

Various cell populations and the amount of cell stress (in terms of stressor moieties or units) at time *T* = 0 and the value of *Ψ*[Rx] = ±d[Rx]/d*k_i_* at *T* = *X* years which ranges from 1 to 20 have been summarized in Table 1. The findings summarized in Table 1 correlate these cell populations with their interaction coefficients with each stressor moiety. It is evident from the table that microglial sensitivity/activation correlates highly with RGC death whereas quiescent microglia negatively correlate with RGC death indicating that migroglial dynamics has a strong correlation coefficient with RGCs survival and demise.

The solutions to these equations yield an intriguing amount of information about glaucoma pathogenesis and neuron–glia interactions. The basic premise of this study was to ensemble all the list of pathogenic processes in glaucoma into two basic outcomes, described as RGC death and survival. A combination of the processes and their kinetic factors is shown in Figure 1. Whether an RGC dies or survives in a particular set of conditions would depend on its sensitivity which is individual-specific and depends on a number of factors describable in terms of our sensitivity equation. Elaboration of our sensitivity equation describes certain important features of glaucoma including normotensive glaucoma and nonglaucomatous ocular hypertension. Though there are many factors that describe this sensitivity (*viz.,* inflammatory response, inter- and intracellular signaling, oxidative stress, insulin signaling and energy metabolism, apoptosis, electrolyte and ion homeostasis, synaptic neurotransmission, protein misfolding, intraocular pressure, intracranial pressure, mitochondrial integrity, and genetics), they can ideally be represented by a sensitivity coefficient (*Ψ*). This sensitivity coefficient when described in terms of the 7 rate equations tends to predict the etiogenic threshold and the rate of disease progression.

Out of the 22 processes identified by the model, 15 are physiological and are involved in the maintenance of homeostasis whereas the remaining 7 are pathological and override the 15 physiological homeostatic processes once the glaucoma pathology is initiated. This indicates that the kinetic parameters of these processes are the deciding factors of glaucoma onset and progression. A combination of all these 22 processes with their kinetic properties can be employed as the predictive factor for glaucoma risk, onset, and rate of progression.

The results of the rate equations with respect to temporal loss of RGCs are given in Table 1 whereas Table 2 gives a description of the sensitivity of each cellular event in glaucoma pathogenesis. These results indicate that inflammation is one of the earliest markers for glaucoma, ensuing long before any other symptoms appear. A close look at these tables indicates microglia to be the most potent target for glaucoma as suggested by the sensitivities and the even rate kinetics.

**Table 2:** The sensitivity and efficacy of each cellular event involved in glaucoma pathogenesis. This table shows the potential pathways that can serve as early intervention targets in glaucoma

*Pathway*	*Rate*	*Pathway efficacy*
Aα → Rσ	*k*_1_	Very high
Aβ → Rδ	*k*_2_	Low
Mβ → Rδ	*k*_3_	Low
Mα → Aα	*k*_4_	Low
Mβ → Aβ	*k*_5_	Low
Rσ → Mα	*k*_6_	Low
Aα → Mα	*k*_7_	Very high
Cσ → Mα	*k*_8_	Moderate
Mβ → Mα	*k*_9_	Low
Rδ → Mβ	*k*_10_	Low
Rσ → Mβ	*k*_11_	Moderate
Aα → Mβ	*k*_12_	Very high
Cσ → Mβ	*k*_13_	Moderate
Mα → Mβ	*k*_14_	Very high
Rσ → Cσ	*k*_15_	Low
Mα → Cσ	*k*_16_	Low
Gα → Rσ	*k*_17_	Very high
Gβ → Rδ	*k*_18_	Low
Mα → Gα	*k*_19_	Low
Mβ → Gβ	*k*_20_	Low
Gα → Mα	*k*_21_	Very high
Gα → Mβ	*k*_22_	Very high

Eqn (VI) indicates that the RGC survival is inversely related to the activation of microglia. Since the kinetics of the equation suggests irreversibility, it indicates that microglial activation may be a pivotal event in RGCs population depletion in glaucoma. This equation suggests a microglia-mediated switch in RGC priming for apoptosis. This indicates that glaucoma pathogenesis follows a two-hit model involving the first hit with priming for apoptosis and the second hit for an irreversible decision for RGC demise in apoptosis.

Eqn (VI) also indicates that an interaction coefficient of microglia with Muller cells does not exist and, therefore, these cell types may or may not exchange imperative functional signals in physiological and inflammatory conditions. In terms of the diffusion coefficient, this equation also describes the usefulness of anti-inflammatory factors in bringing down cell stress independently in these two cell types. This equation may also be employed in simulating clinical trials of the drugs that hold potential to address inflammation in glaucoma.

## DISCUSSION

The present study is the first mathematical model developed for neuron–glia interaction in glaucoma. This model emphasizes that the long-ignored field of systems biology and mathematical modeling approach is essential to supplement the conventional experimental studies in order to realize the long-sought goals for the development of effective treatment for glaucoma. Due to the multiple levels of etiopathogenesis and involvement of a great number of processes, the interaction becomes complex with multiple nodes which need to be understood in an all-inclusive approach with network level reach and high precision. Conventional experimental protocols are likely to be less successful if not guided by system-level studies. A combination of experimental studies with mathematical modeling is likely to improve the outcome of experiments and help in getting better insights.

Microglia are one of the most important form of supportive tissue of the optic nerve. Glial cells are 5 to 10 times more in number than neurons. The blood–brain barrier and the blood–retinal barrier restrict the entry of immune cells from the blood to the central nervous system and retina. Microglia are one of the main immune defense of retina and are involved in degeneration of the RGCs. Microglia play an important role in cellular apoptosis and removal of dead cells, and therefore, their function has a direct effect on cellular density in the RGC layer which is a measure of the disease progression in glaucoma. Our model suggests that microglial activation is one of the earliest events in glaucoma which is in agreement with the previously published literature.^21^ Our model, therefore, has direct clinical application and neuroprotective regimens aimed at inhibiting microglial activation are likely to increase the RGC density and therefore availability of RGCs for neurorestoration and neuroprotection. Inhibiting microglial activation may, according to our results, lead to a decrease in the rate of depleting RGCs in the retina. The present mathematical approach provides a justification for novel treatment modalities aimed at inhibiting the RGCs scavenging by microglia.

By destroying the dying cells, microglia have a role in clearing the debris. But in this disguise, a significant cell population may be lost which is otherwise available for regeneration and restoration. Interestingly, one of the neuroprotective functions of microglia is to secrete insulin-like growth factor-1.^22^ This is in support of “The Brain Diabetes Theory of Glaucoma.” Over-reactive microglia are detrimental to surviving cells as well as the ailing RGCs. They lead to the formation of multiple inflammatory neurotoxic factors such as TNF-α and inducible nitric oxide synthase (iNOS).^23^ This is particularly relevant to the optic nerve head (ONH) as TNF-α expression shows an upsurge in perivascular and parenchymal microglia in the ONH area. iNOS has also been reported to be elevated in the ONH of glaucoma patients^24^ very early in the glaucomatous process which lends support to our findings in the present mathematical model.

Inhibition of microglial activation is already an area of active research which imbibes further support from our findings. Our mathematical model, therefore, supports the premise of the protective effects of minocycline (a tetracycline derivative) which may prove to be a useful neuroprotective agent in glaucoma by working to keep the RGC density high. As per this logic, minocycline may also have a preventive value in glaucoma. This finding is in agreement with the already published reports.^25–29^ Since microglia function as immune cells of the CNS, anti-inflammatory agents can be potent candidates for research in glaucoma therapy. Triamcinolone acetonide and amcinonide (corticosteroids), thought to be inhibitors of microglial activation, may provide candidature for glaucoma therapy.^30^ These agents also inhibit TNFα and iNOS production thereby lending support to the findings of the present mathematical model. Not only this, many herb extracts, such as ginsenosides,^31^ curcumin,^32^ gingerol,^33^ citicoline,^34^ quercetin,^35^ etc., have also been found to inhibit microglial activation. In addition, insulin^36^ and metformin^37,38^ also suppress microglial activation thereby lending support to “The Brain Diabetes Theory” of glaucoma.^2,3,11^

## CONCLUSION

The proposed mathematical model may be useful in understanding the pathogenesis and neuron–glia interaction in glaucoma. It also indicates that prevention of glial (particularly microglial) activation may form the early and effective treatment regimens for preventing and treating glaucoma. Our proposed model suggests that inhibiting microglia in glaucoma may keep the RGC density in the retina high and may therefore increase the availability of RGCs (nevertheless ailing RGCs representative of the grey areas in the visual field) for restoration. Modern treatment approaches for glaucoma should consider this concept in addition to the regular regimen of entirely focusing on bringing down the intraocular pressure in glaucoma patients. The results of the present mathematical model also suggest a need for identification of therapeutic regimens that will specifically inhibit activation of retinal microglia thereby raising hope for prevention of progressive damage as well as neurorestoration in glaucoma.

The etiopathomechanism of glaucoma involves many moieties in addition to RGCs and glia. The present mathematical model is based on the 22-event understanding shown in Figure 1. Since the model comes up with rate equations with respect to glial activation and cellular stress and consequent apoptosis, this model can be used to investigate the efficacy of drugs that might present a candidature to slow down the disease progression.
